# Transformative, interdisciplinary and intercultural learning for developing HEI students’ sustainability-oriented competences: a case study

**DOI:** 10.1007/s10668-022-02208-7

**Published:** 2022-03-01

**Authors:** Mélodine Sommier, Yijing Wang, Ana Vasques

**Affiliations:** 1 Department of Language and Communication Studies, University of Jyväskylä, Jyväskylä, Finland; 2 Department of Media and Communication, Erasmus University Rotterdam, Rotterdam, Netherlands; 3 Erasmus University College, Erasmus University Rotterdam, Rotterdam, Netherlands

**Keywords:** Urban sustainability, Pedagogical tools, Transformative learning, Interdisciplinary, Intercultural education, Course design

## Abstract

The literature has produced relevant theoretical insights into pedagogical frameworks, tools and competences that would be best suited to teach sustainability at higher education (HE). This article contributes to such a discussion using a course on sustainability developed by us as a case study. Two research questions are tackled in this article: (1) How to empower students to address urban sustainability challenges through the inclusion of transformative, interdisciplinary and intercultural learning into the current HE system? (2) Which pedagogical tools can be used to develop students’ sustainability-oriented competences? To address the research questions, the case study consists of two parts. First, by reflecting on the course design, this article aims to shed light on the benefits and challenges of transformative pedagogy and of an interdisciplinary and intercultural framework. Second, by analyzing students’ learning diaries (*N* = 36) using thematic analysis, this article offers insights into some of the students’ learning process, allowing us to assess the strengths and weaknesses of the course design as well as draw implications to improve and renew courses on sustainability in HE. The findings from the learning diaries indicate the students’ thirst for formal knowledge on sustainability, which they connected to their professional development and yearning for action. The learning diaries also suggest students’ increasing awareness of sustainability as a systemic and structural issue during the course, which aligns with the transformative learning framework used. Finally, this study emphasizes the need for structural support to meaningfully integrate sustainability in HE curricula and teaching practices.

## Introduction

While urban sustainability has developed into a contested societal discourse which calls for higher education (HE) to produce creativity, critical thinking and sustainability-oriented competencies, current HE system does not provide many opportunities to achieve these goals (Scharmer, 2018; Yanez et al., 2019). The literature has produced relevant theoretical insights into pedagogical frameworks, tools and competences that would be best suited to teach sustainability at HE (e.g., Evans, 2019; Lozano et al., 2017; Remington‐Doucette et al., 2013; Rieckmann, 2017; Wiek et al., 2011). However, scarce research has examined the connection between pedagogical tools that foster sustainability-oriented education competencies and the students’ learning experiences. This article contributes to such a discussion using a course on sustainability that we developed as a case study and whose pedagogical design (i.e., transformative, interdisciplinary and intercultural setup) was rather unique.

The path toward sustainability is inherently linked to the ability to overcome complex and multifaceted problems that have no clear straightforward solutions. It is widely acknowledged that sustainability education requires the transcendence of the aims and methods of single disciplines (Jones et al., 2010; Lam et al., 2014). Complex problems have no clear boundaries and, thus, a mono-disciplinary approach is often inadequate to address such issues (see, e.g., Annan-Diab & Molinari, 2017; Gantogtokh & Quinlan, 2017). A shift to interdisciplinary education might require, however, considerable adjustments from both the teachers and the students involved as well as from HE institutions. Teachers should be able to promote a dialogue between different perspectives, discourses and methods of addressing sustainability issues, which requires an open attitude and the willingness to learn while engaging with different viewpoints (Feng, 2011). On the other hand, the challenges that students might feel in an interdisciplinary setting can be linked to the rapid switch between disciplinary domains and to the disciplinary jargon that is often used (Abbonizio & Ho, 2020). The advantages and challenges of interdisciplinary courses resemble those of intercultural education. The development of intercultural competencies is permeated with complex processes as individuals engage with perceived cultural differences (Dervin, 2016). Embedding our course on sustainability in an interdisciplinary and intercultural framework was an opportunity to enhance its transformative orientation. It allowed us to explore obstacles and incentives for action and to engage with sustainability issues from a holistic multifaceted perspective. This transformative orientation aimed at empowering students to become sustainability competent and actors of positive change (Frisk & Larson, 2011; Schnitzler, 2019).

A somewhat unique aspect of the course discussed in this article was our different academic backgrounds (intercultural communication, environmental sciences and business and society management). During course development, we had to cultivate an open mindset in order to find understanding, connections and a common discourse before agreeing on the contents and methodologies used in the course. This co-creation process also helped us lessen the barriers for students with diverse backgrounds.

By reflecting on the course design (part I), this article aims to shed light on the benefits and challenges of transformative pedagogy and of an interdisciplinary and intercultural framework. In addition, findings from the students’ learning diaries (part II) offer insights into some of the students’ learning process, allowing us to assess the strengths and weaknesses of the course design as well as draw implications to improve and renew courses on sustainability in HE. Two research questions (RQ) are tackled in this study specifically.

**RQ1**: How to empower students to address urban sustainability challenges through the inclusion of transformative, interdisciplinary and intercultural learning into the current HE system?

**RQ2**: Which pedagogical tools can be used to develop students’ sustainability-oriented competences?

## Methodology

Using one course as a case study, this article presents solutions as well as avenues for improvement to teach sustainability in HE. We first reflect on the course structure, pedagogical tools and learning goals and then analyze the students’ experiences as expressed through their learning diaries. All of the students enrolled in the course were informed about the study and invited to give consent for their learning diaries to be used as data. Half of the students gave their consent (12/24) allowing us to analyze their 36 learning diary entries.

**Fig. 1:**
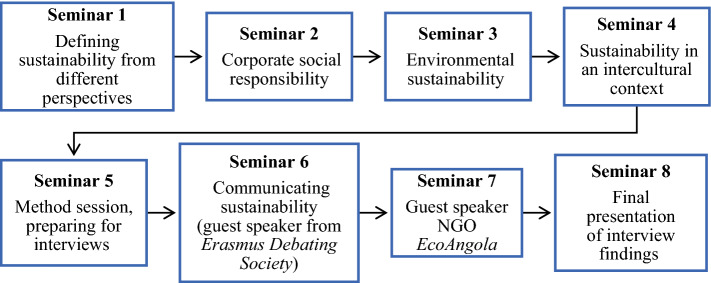
The structure of the honors course on sustainability

The honors course (i.e., an extracurricular course for highly motivated students) took place in April–May 2020 and was conducted over an 8-week period during which students attended a weekly three-hour online seminar (24 h), read required literature (20 h), worked on individual reflection assignments (24 h) and conducted group work for a final research project (72 h). Twenty-four students from six faculties were enrolled (see Appendix 1) and selected based on the short motivation letter they wrote when applying to the course. The students’ small-scale research project consisted of collecting and analyzing interviews regarding urban sustainability in China, Ecuador and the Netherlands. The first four weeks of the course were designed to provide students with sufficient knowledge to dive into their research projects. During the second half of the course, students worked in groups to conduct interviews with participants from the three countries. The findings were analyzed in groups and presented during week 8. At the end of the course, students recorded a short video sharing their understanding of sustainability challenges and possible solutions based on the interview findings and course content. The structure of the course is presented in Fig. 1. The course was well received by the students who filled in the anonymous final course evaluation (overall grade of 5.4/6).

Three curricular positions proposed by Alvarez and Rogers (2006) were integrated into the course design, referring to the *definitions of sustainability*, *implementation of sustainability*, and *sustainability as discourse*. The first curriculum position—the *definitions of sustainability*—raises three questions: (1) “where they have emerged from”; (2) “what they attempt to achieve”; (3) “how they can be compared” (Baker, 1997, cited in Alvarez & Rogers, 2006). These questions were not only the focus of the discussion in seminar 1, but also constantly revisited throughout the course. Sustainability was defined in a bottom-up manner so students could uncover the different meanings assigned to this term across national, cultural, discipline-specific and historical contexts. In that sense, the *definitions of sustainability* were indissociable from *sustainability as discourse*, a topic that is situated, contested and evolving.

The second curriculum position—the *implementation of sustainability*—draws on three aspects, which are (a) “what is unsustainable”; (b) “how to make practices more sustainable”; (c) “how to evaluate sustainable outcomes” (Alvarez & Rogers, 2006). Along with this position, seminars 2, 3 and 4 were designed to address important indicators for assessing sustainable activities and their consequences, such as the triple bottom line (TBL), ecological footprints and doughnut economics.

The third curriculum position focuses on *sustainability as discourse*. The design of seminars 5, 6, 7 and 8 is aligned with this focus, which emphasized the complex and situated conceptions of sustainability. Through interviewing participants from three countries (China, Ecuador and the Netherlands), engaging in an online debate about pressing sustainability issues, and dialoguing with the founder of an NGO (EcoAngola), students succeeded in acquiring an understanding of sustainability as a contested discourse utilized by competing individuals, groups and across cultures. Moreover, they started to critically reflect on their own behaviors and proactively propose possible changes which needed to take place.

The integration of the three curricular positions allowed us to overcome current sustainability challenges in HE system, such as common misconceptions of sustainability as only related to technology, disconnected from arts, humanities and social science disciplines (AHSS), with little relevance in social and attitudinal aspects, or as a concept barely involving complex issues (Seatter & Ceulemans, 2017). Also, as the three curricular positions complement each other, the course provided the students a steady and systematic way to examine the multilayered notion of sustainability.

Analyzing the students’ learning diaries can offer insights into the effectiveness of such a course design. The students’ narratives about the course might clarify how students perceived their learning experiences, how they connected features of the course to their learning journey, and how their understanding of sustainability developed. In total, three learning diaries were included in the course with guiding questions (see Appendix 2) that invited the students to critically engage with specific aspects of the course content and reflect on their level of awareness. Students were informed from the start that taking part in this research was voluntary, reversible at any point, and would have no effect at all on their grades in the course. Only the learning diaries of the students who gave their consent (i.e., 12/24) were analyzed for this study.

In the first step, the learning diaries were anonymized and catalogued. Next, the 36 (i.e., three per students) learning diaries were analyzed using thematic analysis and the three coding steps outlined by Boeije (2009). In the open coding phase, elements from the learning diaries about the students’ previous and current knowledge of sustainability, the students’ learning goals, the students’ reflection on the learning process, course content and course design, as well as on their positionality as learners and citizens were coded. In the axial phase, emerging findings were outlined to try and identify patterns as well as notable exceptions. These emerging findings were refined by regularly going back to the data to confront the researchers’ perceptions to the students’ narratives. Academic literature, anonymous course evaluation and materials from the course (i.e., syllabus, assignments and slides) were used to compare, contrast and challenge the developing analysis. Finally, following from this iterative process of navigating between data, academic literature and emerging findings, we identified and outlined two main themes: *the importance of the group* and *the thirst for knowledge and action.* The findings are presented in Part II of the result section and used to reflect on the course design as well as structural issues in HE. Part I of the result section offers an in-depth reflection on the course design and the three pillars around which it was structured.

## Results

### Part I: reflection on the course design

To allow the students to understand sustainability as a contested discourse used by competing individuals and groups, and across cultures, we embedded three pillars in the course design, which are *transformative learning as the overarching orientation, interdisciplinary and intercultural education at the core,* and *integrating pedagogical tools for developing sustainability-oriented competences*.

#### Pillar I: transformative learning as the overarching orientation

Sustainability being a contested discourse demands the facilitation of critical thinking and actions (Seatter & Ceulemans, 2017) as well as employing creativity as a means to enable a shift in mindset (Luzano, 2014). Transformative learning fulfills these roles, and, hence, was chosen as the overarching orientation of the course.

According to Seatter and Ceulemans (2017), transformative learning plays a crucial role in devising effective HE for sustainable development. Compared to traditional education which mainly prepares the latter for acceptance of the status quo (Shor, 1993), transformative learning emphasizes developing autonomous thinking through asking critical questions and searching for new sources and ideas (Mezirow, 1997). It not only enables the learners to make their own interpretations of sustainability issues and challenges, but also stimulates them to change their position on “how best to be sustainable” (Seatter & Ceulemans, 2017, p. 55).

Viewing transformative learning as a process, Seatter and Ceulemans (2017) discovered three important outcomes of it. First, through questioning taken for granted frames of reference, students can develop critical thinking to become more open and reflective (Greene, 2001). Second, not only may a shift of mindset occur, but other major changes in acting, relating and being can also take place during the process (Bennetts, 2003). Third, transformative learning allows the students to assess their own performance and evaluate values for their effectiveness toward shared sustainable goals (Bhaskar, 2009, cited in Seatter & Ceulemans, 2017). In summary, we chose transformative learning as the overarching orientation of the course to increase students’ awareness of biases and assumptions connected to sustainability.

To achieve the overarching goal, we adopted a constructionist and learner-centered approach as our teaching philosophy. It required the instructors to actively involve students in constructing knowledge for themselves and developing new ideas based on their current knowledge and past experiences (Nie & Lau, 2010; Tenenbaum et al., 2001). In other words, rather than behaving as passive learners, students needed to involve themselves in the continuous process of constructing, negotiating and reconstructing meanings. As instructors, on the other hand, we served as facilitators—not a sage on the stage, but a guide on the side—who invited and encouraged students to activate their prior knowledge, participate in discussions and debates, embrace critical thinking and construct knowledge for themselves.

#### Pillar II: interdisciplinary and intercultural education at the core

In addition to the transformative and constructionist bases on which the course design drew, the course also incorporated two main dimensions that we perceived to be intrinsically connected to sustainability: interdisciplinarity and interculturality.

Interdisciplinarity as knowledge regime is critically demanded to address complex problems (Felt et al., 2013). The need is even more compelling in our era with increasingly complex sustainability challenges. Interdisciplinarity implies a process of integration of different insights, values and fields of knowledge, for example between the arts, humanities, social sciences, among themselves and with science, technology, engineering, mathematics and medicine. Such process aims at building a common ground and an overarching understanding of a problem (Holley, 2009; Porter et al., 2006), which is particularly relevant to sustainability education (Baumber, 2021).

A key driver for interdisciplinary education is defined in social science. The literature argues that the opportunity for learning lies in the boundary of disciplinary, cultural and social groups (Gantogtokh & Quinlan, 2017; Klaassen, 2018). A “third space” can be created at these boundaries, in which the meeting of different perspectives triggers the co-construction process of learning (Akkerman & Bakker, 2011; Almasi, 2016). In particular, the third space is often realized in overarching thematic areas such as sustainability, which stimulates learners to become open and critical about different perspectives and develop new ideas (Akkerman & Bakker, 2011). It implies that the realization of the third space requires “a high level of breadth and complexity in the problem” (Klaassen, 2018, p. 843).

To help students acquire an interdisciplinary understanding of sustainability, we integrated multiple sources of knowledge, methods and perspectives from two or more disciplines in the course. The fact that we came from different disciplines allowed us to encourage the co-construction of learning by approaching complex sustainability issues from the ecology, communication and management perspectives, while synthesizing knowledge from these fields with a critical lens. In other words, the process of integrating different insights and values allowed the realization of the third space in the course. Moreover, the students who were admitted to the course held diverse academic backgrounds (see Appendix 1). Blending them in the study further strengthened the interdisciplinary orientation of the course as the heterogeneity of their prior knowledge, understanding of sustainability challenges and skills to approach and address a problem all contributed to the co-construction process of learning.

Intercultural education is recognized as a strategic resource toward sustainability and social cohesion (Salgado-Orellana et al., 2019). The importance of intercultural education has been reinforced by processes of globalization and increased emphasis placed on diversity (Sorkos & Hajisoteriou, 2020). Among the 17 Sustainable Development Goals of the UN, Goal 4 specifically states the need to ensure inclusive and equitable quality education, so that all individuals can fully develop their potential (United Nations, n.d.). Along with this goal, we consider developing students’ intercultural competences, which allows them to interact and cooperate with peers they perceived to be of different social and cultural backgrounds, as an important learning outcome of the course.

Intercultural education not only deals with the interplay between global and local, but also the importance of constantly navigating between both rather than choosing one over the other. It offers a systemic perspective by exposing the interconnectedness of different layers, including people, micro- and macro-level structures, as well as past and present discourses (Faas et al., 2014; Holliday, 2018). In addition, as it entails the analysis of socially constructed reality (Holliday, 2018), the power relations, ideologies and struggles embedded in discourses and practices about sustainability can all be addressed in intercultural education. As interculturality embraces the complexity of social relationships and realities (Dervin, 2016), it is very well suited to address the “volatility, uncertainty, complexity and ambiguity” (VUCA) of the world. Intercultural education serves as a mirror for students to reflect on their own (sustainability) practices and assumptions, and the political, historical, economic structures in which these are embedded.

The interculturality position of our course is reflected in several aspects. First, when selecting students for the honors course, we took interculturality into account which ensured a diverse group of students in terms of gender, nationality and academic background. Second, we dedicated one seminar to address the intercultural perspective on sustainability. During the seminar, we developed a “mapping sustainability” activity to examine how sustainability as a global issue is related to local contexts, and how our knowledge of sustainability is shaped by ethno- and Western-centric discourses. Third, using debate as a pedagogical activity (see Table 1), we confronted students with different opinions, thus encouraging them to critically question the ideological dimension of discourses about sustainability and to engage with the VUCA of the world and its implications. Forth, non-Western views were extensively discussed throughout the course and well incorporated into the cross-cultural research design of the hands-on research project.

**Table 1:** Pedagogical tools adopted in the course for developing sustainability-oriented competences

Classification	Pedagogical tools	Summary	Sustainability-oriented competences addressed
Universal	Case studies	Students considered real-world examples and examined issues from a diversity of stakeholder perspectives. (Example: Students’ had to analyze a current case in detail for their presentations)	Systems thinking; interdisciplinary work; critical thinking and analysis; communication and use of media; assessment and evaluation
	Lecturing	The instructors lectured on key topics in relation to sustainability, such as corporate social responsibility, environmental sustainability, and sustainability in an intercultural context	Anticipatory thinking; justice, responsibility and ethics; empathy and change of perspective
	Flipped classroom	The learner-centered model was taken: Students were initially introduced to new topics through readings and the preparation of activities out of the class. The online sessions were used to explore topics in greater depths and create meaningful learning opportunities	Interdisciplinary work; anticipatory thinking; critical thinking and analysis
	Debates	Students participated in the process involving formal discussions on predetermined motions and learnt to put forward opposing arguments to argue for different viewpoints. (Example motion: This house would abolish all private property to protect the environment.)	Critical thinking and analysis, empathy and change of perspectives; communication and use of media
	Interdisciplinary team teaching	The course integrated the expertise of three instructors lying in ecology, intercultural communication, and business and society management	Systems thinking; interdisciplinary work; tolerance for ambiguity and uncertainty
	Hands-on research	Students conducted interviews with participants based in China, Ecuador and the Netherlands. The research question was predetermined by each group, aiming at understanding and comparing local struggles and sustainability solutions	Systems thinking: interdisciplinary work; interpersonal relations and collaboration; empathy and change of perspective; communication and use of media; strategic action; assessment and evaluation
	Learning diaries (used as data for this research project)	Students submitted three learning diaries, critically reflecting on their learning journey and assessing the knowledge and skills developed throughout the course	Critical thinking and analysis; strategic action; assessment and evaluation
	Mind and concept maps	In the last session, students developed a nonlinear outline of the conceptions of sustainability. Sustainable solutions at the micro-, meso- and macro-levels were represented in the mind map graphically	Systems thinking; interdisciplinary work; critical thinking and analysis; personal involvement
Community and social justice	Jigsaw/interlinked teams	Each group was assigned one concept that they were asked to teach the rest of the class about through their presentations	Interpersonal relations and collaboration; empathy and change of perspective; communication and use of media; strategic action; personal involvement
	Guest lecture	The guest lecture was delivered by the founder of the NGO EcoAngola. Local environmental, economic and social challenges were presented and critically discussed	Systems thinking; interdisciplinary work; empathy and change of perspective; strategic action
Environmental education	Eco-justice and community	Discussion in class had a significant emphasis on environmental racism and class discrimination. In addition, a role play was developed to allow the students to assess eco-justice issues in different regions, such as industrialized nations, newly industrialized nations, former Soviet bloc countries, and less-developed countries	Systems thinking; interdisciplinary work; anticipatory thinking; justice, responsibility and ethics
	Traditional ecological knowledge	Three instructors and the guest lecturer highlighted indigenous knowledge systems and values to help the students understand threatened cultural diversity and heritage, and critically question their own position on how best to be sustainable	Interdisciplinary work; anticipatory thinking; justice, responsibility and ethics; empathy and change of perspective

#### Pillar III: integrating pedagogical tools for developing sustainability-oriented competences

The desired learning outcomes of the course are described through sustainability-oriented competences. Competence-based education can be distinguished from either repetition or indoctrination, as it draws on students’ ability to develop new ideas and address complex issues, as opposed to inculcation of rote habits (Lozano et al., 2017).

Developing some of the competences requires the instructors to combine different pedagogical tools in one education program. While the adoption of a pedagogical approach highly depends on the educational goals and learning environment, the variation in pedagogical approaches adopted also matters importantly given the diversity of students (Ceulemans & De Prins, 2010). The variation not only ensures different learning processes, but also allows the students to enhance their learning capacities and skills (Lozano et al., 2017; UNESCO, 2009).

Table 1 presents the pedagogical tools adopted in the course and their connection with desired sustainability-oriented competences. We followed Lozano et al. (2017) to classify the chosen pedagogical tools into: a) universal, b) community and social justice and c) environmental education approaches and list the sustainability-oriented competences to which they correspond. The summary column offers more concrete insights into the implementation of the pedagogical tool in our honors course.

As shown in Table 1, the course mainly adopted universal pedagogical tools, whereas community and social justice tools and environmental education tools were included as complementary approaches. Some sustainability-oriented competences were addressed through multiple activities, such as systems thinking, interdisciplinary work, critical thinking and analysis, and empathy and change of perspective. But no single pedagogy tool alone covers all competences. It implies that to better shift the mindset and trigger actions of the students, a variety of pedagogical tools need to be integrated for developing sustainability-oriented competences (see, e.g., González-Zamar et al., 2020).

### Part II: findings of the learning diaries

In their learning diaries, students mentioned the *importance of the group* in the learning process. That is, the collaborative dimension of the course which encouraged students to learn from and with each other seemed to be a significant and positive aspect of the learning experience. This connects to the setup of the course and the three pillars discussed above. The emphasis placed on transformative learning highlighted the role played by students in being actors of their learning development as well as supporting others. It is worth noting that the importance of *people* was mentioned even though the course was fully online due to Covid-19. This underlines the importance of not having too large groups (in this case, 24 students) to allow for meaningful interactions and create a safe space. The pedagogical tools (see Table 1) encouraged various types of collaborations and discussions among students and with the teachers. Having small groups of learners is a central aspect to ensure a positive learning experience by allowing for differentiation, ensuring a safe environment and developing competences that are critical to sustainability such as critical thinking and empathy.

The feeling of empowerment associated with peer-collaboration was connected by the students to specific characteristics of the group. Namely, the intercultural and interdisciplinary dimension seemed to play an important role in motivating students to engage with one another. They indeed regularly mentioned how rewarding it was to be in the same course as students and teachers from different Faculties. Interestingly, this was also described in the students’ learning diaries as a unique experience. This highlights an important structural limitation of HE which tends to advocate for interdisciplinary and intercultural education, but often fails to implement such practices (Cole & Meadows, 2013). The emphasis placed by the students on the interdisciplinary and intercultural dimension of the course calls for more integrated teaching practices and interdisciplinary collaboration. This, as we found out when organizing this honors course, requires significant institutional support in order to bring down walls between Departments and Faculties. However, as Filho et al. (2018) rightly point out, despite a discursive emphasis on sustainability, very few Universities provide sufficient institutional support to renew curricula and teaching practices. This jeopardizes the quality of sustainability education that, ultimately, tends to be limited to isolated efforts “in stand-alone courses, often with pedagogies not entirely appropriated to SD principles.” (Filho et al., 2018, p. 287). Structural support is therefore essential to ensure HE produces more than greenwashing discourses.

Institutional support is crucial given the challenges associated with designing an interdisciplinary course. This requires more than presenting different fields of study. It entails searching for and drawing connections between disciplines to show how they can complement one another (Klaassen, 2018). One of the challenges that we encountered was also the need to find an appropriate level of communication (not superficial, but also not too specific) that would allow us, teachers and students of different disciplinary backgrounds, to speak at “the same level.” Another challenge was to adjust to different ways of organizing classes, which we have overcome by using various pedagogic tools (see Table 1) that facilitated critical and respectful exchanges of ideas and allowed students to connect sustainability approaches with their own experiences. This, in turn, has proven very useful in accommodating interdisciplinary views as well as in promoting communication between teachers and students of different backgrounds.

Although the diversity of the group was generally praised by the students, particularly in terms of disciplinary backgrounds, age and personal (international and professional) experiences, a few students mentioned the homogeneity of the group in terms of sustainable interests. Students who applied for this course were all interested in sustainability and motivated to learn about it. In addition, as a highly selective course (~ 28% acceptance rate), students whose motivation translated into extracurricular activities connected to sustainability were more likely to be selected. This means most students held rather similar world views and had a shared understanding of sustainability as a social and political urgency. Some students referred the homogeneity of the group as a positive aspect that strengthened their sense of belonging and contributed to their learning development:

In this course I have met and spoken to a lot of like-minded people, that are far more advanced in the topic than I am and are using their awareness to spread the word and make a change. This has really empowered me to spread more awareness, and I have experienced positive reactions from the people around me. (Student 12)

Being part of a committed and like-minded group can contribute to create a safe learning environment, particularly as sustainability remains a contested topic (Matheson & Sutcliffe, 2017). However, the homogeneity of the group can also limit the learning experience as students are not confronted to a wide variety of viewpoints and experiences. As teachers, we tried to mitigate this limitation by drawing on transformative pedagogical tools and including an empirical research project. By conducting in-depth interviews with participants of different social backgrounds living in different countries (i.e., China, Ecuador and South Africa), students were confronted with varying definitions of and appreciation for sustainability. This empirical project, and the pedagogical framework in which it was embedded challenged what some of the students took for granted and allowed them to go beyond ethno- and Euro-centric views of sustainability:

Some of my misperceptions were also challenged by debates and presentations as I learned that Europe is not as sustainable as it seems to be. This demonstrated some of the complexity of sustainability how it can be approached and measured differently. (Student 9)

Being confronted with different social, national and cultural realities – and associated discourses – revealed to students the complex interplay between global and local levels, particularly as past and present ideologies and power struggles, including colonialism, nationalism or neoliberalism, come into play. Thus, in addition to offering contrasting worldviews, the empirical part and general framework of the course revealed the VUCA of the world and, therefore, of sustainability – as mentioned by some of the students:

I acquired vast amounts of new knowledge regarding sustainability which not only deepened my understanding, but also showed me that the concept of sustainability is far more complex than I initially thought. (Student 5)

The second main theme we identified as prevalent in the students’ learning diaries was their thirst for *knowledge and action*. The students repeatedly addressed their perceived lack of theoretical knowledge about sustainability. The emphasis placed on knowledge was connected to their longing for action. These students wanted to know in order to act and reported a perceived connection between their intellectual awakening and their actions:

Despite having witnessed a lot of environmental damage in my childhood, it is only when arriving in Rotterdam that I had a click and wanted to change my lifestyle radically. This click came after watching the documentaries ‘Cowspiracy’ and ‘What the Health’ by Kip Andersen. I was really shocked by the overload of information that I had never suspected existed. This gave me the drive to do more research (I ended up writing about vegetarianism and climate change in the essay that I had to work on in my class Academic Writing) and I ultimately became a vegetarian two years ago. (Student 10)

Taking this course was often described by the students as a significant step in their sustainable journey. Many of the students explained learning about sustainability late in life (at the University or in High School) and by accident or informally (e.g., incident, documentary and friends). None of the students had learnt about sustainability in lower education and very few of them had received any class on sustainability at the University. Student associations, personal networks and (social) media were the main platforms through which they had developed an understanding of what sustainability entails. Gaining formal knowledge was therefore highlighted as a very important aspect of their personal and intellectual growth, which several students also connected to their future professional goals.

The importance placed by the students on receiving formal scientific knowledge about sustainability, and the lack thereof in their educational trajectory, raises several issues. First, it indicates the confinement of sustainability as a private matter, something that one learns about on their own- if at all- rather than a scientific subject and societal priority embedded in school and university curricula. Second, exclusively learning about sustainability through media discourses and peers is limited and limiting and may provide students with unidirectional and biased views instead of scientific knowledge. Third, the increased motivation mentioned by the students since they had learnt about sustainability, and the connections they drew between knowing and acting hints at the impact that learning can have and, therefore, at the fundamental role that HE is yet to play in provoking change. Fourth, only students who were interested in sustainability prior to the course enrolled in it. The prestige and selective process of honors courses suggests the group consisted of students with a high-grade average and cultural capital. The lack of courses on sustainability in HE therefore maintains sustainability as an extracurricular activity reserved to privileged students, thus ensuring that sustainability is discussed across courses and in an interdisciplinary manner is the key to ensure inclusive sustainable education in HE.

The connections students drew between knowledge and action aligns with the position of the UNESCO. The UN agency emphasizes the role of HE in helping students develop sustainability attitudes and skills that inform decision making for now and for the future and act upon these decisions (UNESCO, 2009). Successful sustainability education in HE should therefore not only result in increased knowledge but also in a stronger ability and willingness to act. At the start of the course, most students already understood sustainability as a political and societal urgency and therefore already connected, even if implicitly, knowing to acting. However, for some students, their sense of urgency seemed to develop during the course, shifting from primarily individual considerations in their first learning diary to placing more emphasis on structural and holistic views in their last entry. The quotes from students 4 and 7 below show how they connected concepts from the course (i.e., systems thinking competence, triple bottom line and sustainable development goals) to their perception of sustainability (competence) as a holistic issue:

I believe that sustainability is a combined effort of actors where each member accepts responsibility. Therefore, the holistic approach of the *systems thinking competence* is crucial to begin deciphering the complex problems still limiting sustainability. (Student 4, their emphasis)

To me, in order to act sustainably competent, *knowledge* of the existing work related to sustainability like the TBL approach and the SDGs [Sustainable Development Goals] is a useful prerequisite because it helps to view the issues connected to sustainability from a more holistic standpoint. (Student 7, their emphasis)

Students seemed to become increasingly aware of the systemic and structural dimensions of sustainability. While they mentioned the need to act individually, they also acknowledged the limitations of that approach and the need to nudge and exert pressure on powerful structures and entities such as government and companies. In their learning diaries, students connected action to a holistic view of sustainability, showing a developing understanding of the way structures are interconnected and action are not/cannot be isolated. The course design supported such learning outcome. In particular, the transformative learning orientation focused on competencies for action (e.g., discussion on environmental citizenship vs nudging and policies to reduce ecological footprints). Moreover, the interdisciplinary and intercultural framework shed light on the way ideologies and discourses are constructed through and constructive of local and global practices.

## Conclusions

The quest to develop sustainable education at HE requires new educational approaches that move away from traditional knowledge transfer toward a competency-oriented setting. In spite of the abundance of literature on sustainability competencies, insights into the approaches to empower students to address urban sustainability challenges and examples of pedagogical tools that foster sustainability-oriented education competencies are still lacking. In this qualitative study, we aimed to fill this gap by reflecting on the course design of a sustainability honors course and analyzing the students’ learning journeys.

First, the orientation of our course for transformative learning was found to be aligned with the students’ *thirst for knowledge and action*. In their learning diaries, they emphasized a need to know in order to act and explicitly connected their intellectual awakening with their empowerment to take action on sustainability challenges. Remarkably, most of the students had not learnt about sustainability before starting their university studies and most of their knowledge on sustainability derived from informal situations. To achieve formal knowledge was therefore stressed as an essential aspect of their intellectual growth, as well as of their future professional goals. Learning about sustainability as a private rather than a scientific matter embedded in school and university curricula has many pitfalls for the quality and credibility of the knowledge acquired. This situation points to shortcomings in the mission of HE which is yet to facilitate the development of interdisciplinary and inclusive sustainable education. Since sustainability education often takes place within the realm of discipline-oriented faculties, facilitating interdisciplinary education requires structural support from HE institutions.

Second, the focus of our course on interdisciplinarity and interculturality stimulated students to question the ideological dimension of discourses about sustainability and to engage with the VOCA of the world. The interdisciplinary and intercultural framework brought insights into how ideologies and discourses are (re)produced, thus contributing to a holistic understanding of the complex interplay between local and global practices. On a practical level, the course relied on integrating universal, community and social justice as well as environmental pedagogical tools, as no single approach covered all of the sustainability-oriented competencies. The interdisciplinary and intercultural framework was therefore embedded in various aspects of the course (content, participants, pedagogical tools), which may have enhanced its success. We suggest that a similar integrated framework should be taken into account when developing HE for sustainability and that necessary associated structural support should be provided to teachers.

The focus of this study on a specific case study offered an in-depth understanding of the connections between the pedagogical framework adopted and the students’ experiences. These findings call for similar pedagogical endeavors to be implemented and analyzed to understand the various learning journeys students experience. This study focuses on the students’ narratives and, in the future, including teachers’ experiences would be valuable to gain a holistic understanding of the challenges and benefits of implementing transformative, interdisciplinary and intercultural pedagogical tools for sustainability education. In general, the limited scope of this case study invites further research across educational and national contexts to compare, contrast and add to the findings presented here. Although the findings of this research are limited to the case study it focused on, they provide important implications for developing HE for sustainability. The findings are indeed of particular relevance to HE teachers interested in developing sustainability courses embedded in transformative, interdisciplinary and intercultural pedagogy. Given the emphasis placed on the structural support needed to implement such courses, this article is also of relevance to HE decision makers.

## Appendix 1

An overview of the course participants.

**Table:**

Gender	International experience	Faculty	Program	Study level
F	India	RSM	Public Administration	BA-3
F	Italy, Nicaragua and El Salvador	ESSB	Psychology	BA-2
F	South East Asia and Brazil	RSM	MSc International Management/CEMS	MSc
F	Portugal, Angola, United Arab Emirates	ESSB	International Bachelor of Psychology	BA-3
F	NA	ESSB	International Psychology	BA-2
F	Slovakia	ESSB	Management of International Social Challenges	BA-2
F	United Arab Emirates, Japan and Qatar	RSM	Global Business & Sustainability	MSc
F	Germany, Ireland and Japan	ESSB	International Bachelor of Psychology	BA-3
F	Ecuador	ESSB	Bachelor in Management of International Social Challenges	BA-2
F	South Africa and US	RSM	Msc Finance & Investments	MA
F	Germany and Kurdistan	ESSB	International Bachelor of Psychology	BA-3
F	Canada	ESSB	International Bachelor of Psychology	BA-3
F	Brazil and Colombia	RSM	BSc in International Business Administration	BA-3
F	NA	EUC	Liberal Arts & Sciences—Sustainability Major	BA-3
F	South America and Asia	ESHCC	Media Studies	MA
M	NA	ESE	International Bachelor in Economics and Business Economics	BA-3
M	Brazil	ESE and ESPhil	Double Degree Economics and Philosophy	BA-3
M	Japan	RSM	International Business Administration	BA-2
M	China	ESHCC	Pre-master of media and creative industries	Pre-master
M	Ecuador	ESSB	Management of International Social Challenges	BA-3
M	Finland	ESHCC	International Bachelor Arts and Culture Studies	BA-2
M	China and UK	ESSB	Psychology	BA-3
M	NA	RSM	Public Administration/Bestuurskunde	BA-3
M	Two continents	ESSB	Management of International Social Challenges	BA-2

Gender (*F* Female and *M* Male), International experience reported by the students (countries or world regions where the student lived, excluding the Netherlands; NA- the information is not available), Faculty (*RSM* Rotterdam School of Management, *ESSB* Erasmus School of Social and Behavioural Sciences, *EUC* Erasmus University College, *ESHCC* Erasmus School of History, Culture and Communication, *ESE* Erasmus School of Economics and *ESPhil* Erasmus School of Philosophy), program of study and level of study (*BA* Bachelor, *MA* Master, *MSc* Master of Science and year of study when applicable).

## Appendix 2

### Instructions for the learning diaries

Assignment: Learning diary 1

Please answer the following questions in your learning diary:

- How would you define the concept of sustainability?
- What are your main learning goals for this course?
- Explain what role does sustainability play in your everyday life? How does sustainability relate to your identity, if at all?
- This paper is an individual reflection and does not require references or concepts to be used. It should focus on your own perceptions, experiences and ideas.

Assignment: Learning diary 2

- Select two concepts seen in class and discuss how they have contributed to your understanding of “sustainability.”
- Explain these two concepts precisely enough. Go back to the definition of sustainability you gave in your first learning diary and explain how the two concepts you selected have challenged and/or reinforced your initial understanding of sustainability.
- This paper should be minimum 500 words and maximum 800 words.

Assignment: Learning diary 3

- Provide your own definition of “sustainable competence” using at least 2 sources from (the course) literature. Pay particular attention to the knowledge, skills and attitudes that you think should be part of one’s sustainable competence.
- Reflecting on your learning journey, explain whether and how this definition applies to you. Describe the knowledge, skills and attitudes that you feel have been challenged and/or strengthened during the past 8 weeks, and what is still missing in your opinion.
- This paper should be minimum 500 words and maximum 800 words.
